# Heat Treatment of Geopolymer Samples Obtained by Varying Concentration of Sodium Hydroxide as Constituent of Alkali Activator

**DOI:** 10.3390/gels8060333

**Published:** 2022-05-26

**Authors:** Ljiljana Kljajević, Miloš Nenadović, Marija Ivanović, Dušan Bučevac, Miljana Mirković, Nataša Mladenović Nikolić, Snežana Nenadović

**Affiliations:** 1 Department of Materials, “Vinča” Institute of Nuclear Sciences—National Institute of the Republic of Serbia, University of Belgrade, Mike Petrovića Alasa 12–14, 11000 Belgrade, Serbia; marija@vinca.rs (M.I.); bucevac@vinca.rs (D.B.); miljanam@vinca.rs (M.M.); msneza@vinca.rs (S.N.); 2 Department of Atomic Physics, “Vinča” Institute of Nuclear Sciences—National Institute of the Republic of Serbia, University of Belgrade, Mike Petrovića Alasa 12–14, 11000 Belgrade, Serbia; milosn@vinca.rs; 3 Department of Nuclear and Plasma Physics, “Vinča” Institute of Nuclear Sciences—National Institute of the Republic of Serbia, University of Belgrade, Mike Petrovića Alasa 12–14, 11000 Belgrade, Serbia; natasa.nikolic@vinca.rs

**Keywords:** alumosilicate gel, thermal treatment, sanidine, TGA/DTA, compressive strength

## Abstract

In this paper, raw natural metakaolin (MK, Serbia) clay was used as a starting material for the synthesis of geopolymers for thermal treatment. Metakaolin was obtained by calcination of kaolin at 750 °C for 1 h while geopolymer samples were calcined at 900 °C, which is the key transition temperature. Metakaolin was activated by a solution of NaOH of various concentrations and sodium silicate. During the controlled heat treatment, the geopolymer samples began to melt slightly and coagulate locally. The high-temperature exposure of geopolymer samples (900 °C) caused a significant reduction in oxygen, and even more sodium, which led to the formation of a complex porous structure. As the concentration of NaOH (6 mol dm^−3^ and 8 mol dm^−3^) increased, new semi-crystalline phases of nepheline and sanidine were formed. Thermal properties were increasingly used to better understand and improve the properties of geopolymers at high temperatures. Temperature changes were monitored by simultaneous use of thermogravimetric analysis (TGA) and differential thermal analysis (DTA). The loss of mass of the investigated samples at 900 °C was in the range of 8–16%. Thermal treatment of geopolymers at 900 °C did not have much effect on the change in compressive strength of investigated samples. The results of thermal treatment of geopolymers at 900 °C showed that this is approximately the temperature at which the structure of the geopolymer turns into a ceramic-like structure. All investigated properties of the geopolymers are closely connected to the precursors and the constituents of the geopolymers.

## 1. Introduction

Geopolymers are kinds of inorganic polymers that have been based on alumino-silicate materials manufactured commonly at temperatures below 100 °C [1]. Additionally, they have manifested as porous materials of favorable properties, thermal and chemical stability, as well as wide utilization [2]. Potential source materials for the synthesis are natural Al-Si minerals and different wastes. Unlike conventional organic polymers, glass, ceramics, or cement, geopolymers are formed at low temperatures and are heat-resistant, non-flammable, and resistant to strong acids [3]. Geopolymers have become an increasingly popular research area in recent years owing to their good mechanical properties, excellent corrosion resistance and durability, which are especially important for application under high temperatures. In addition, a wide source of raw materials [1,2,4] and low energy consumption make geopolymers an inexpensive material that could be a potential substitute for Portland cement. In order to make geopolymers an alternative to Portland cement, it would be essential to use waste pozzolanic materials with a high content of alumina and silicon dioxide as raw materials for obtaining geopolymers [4]. Geopolymers are widely used for the absorption of toxic organic or inorganic chemical wastes as well as for the fabrication of lightweight and foam concrete [5].

Although extensive research on geopolymers has already been carried out, the development of geopolymers as thermal insulators has not been explored sufficiently [6]. Besides their excellent durability [7,8], the most important property of these materials is their ability to develop high mechanical strength in a short period of time and at a moderate temperature (T < 100 °C) [9,10,11]. It is known in the literature that the geopolymers are superior to Portland cement at 600–800 °C, and even at higher temperatures. Geopolymers are known to be dimensionally unstable in the temperature range of 600–800 °C. Their bending strength is low, as opposed to the compressive strength, which begins to increase slightly after this temperature range. These properties are a consequence of partial sintering, which begins to take place in some of the geopolymer systems at a temperature interval of 600–800 °C, but occurs at slightly higher temperatures [12,13,14,15]. C. Kuenzel et al. 2013 [16] concluded that the compressive strength, porosity, and microstructure of geopolymer mortar samples were not significantly affected by temperatures up to 800 °C. According to some studies, the temperature of 900 °C is the transition temperature for metakaolin-based geopolymers. At this temperature, the geopolymer partially melts. The coagulation process takes place locally, so there are the interspaces that separate locally melted structures. The effect of a change in the silica/alumina ratio on the thermal stability of metakaolin geopolymers shows that all samples showed a decrease in compressive strength after thermal treatment up to 900 °C [17]. Additionally, the concentration of an alkali activator affects the development of the structure of geopolymer materials [18].

Research on the thermal properties of geopolymers has been the subject of many research groups. In this regard, and based on previous research [9,17,19], the objectives of this paper can be presented as the analysis of thermal properties in terms of mass loss and thermal deformation of geopolymer samples exposed to 900 °C. The microstructure and evaluation phase as well as compressive strength properties of thermally treated geopolymer samples are investigated. The main goal of this research is to present the structural and mechanical properties of geopolymer materials as potential ceramic materials.

## 2. Results and Discussion

### 2.1. TGA/DTA Analysis

**Figure 1:**
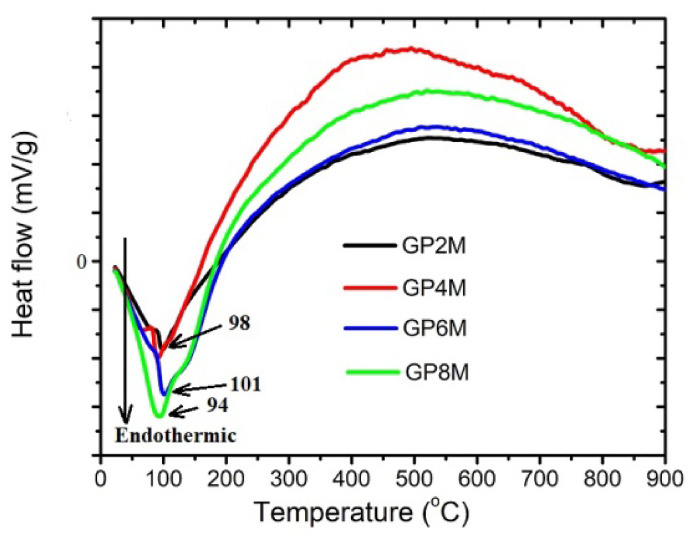
DTA of the reference geopolymer samples GP2M–GP8M.

In order to assess the thermal behavior of GP2M–GP8M samples, simultaneous TGA and DTA were performed. Figure 1 shows the DTA curves of GP2M–GP8M samples. DTA peaks are a consequence of the dehydration process of surface-bound water as well as water molecules in the structure of geopolymer samples. Hygroscopic water forms that exist in geopolymers are removed at a temperature of 120 °C. The width of the peak indicates a possible overlap in the removal of hygroscopic and crystalline water [20]. Chemically bound water begins to be removed after the temperature exceeds 300 °C. Figure 1 shows characteristic endotherm peaks at low temperatures (90 to 200 °C) and a very wide area in the temperature region of 200 to 850 °C, the curves resembling parts of a parabola.

**Figure 2:**
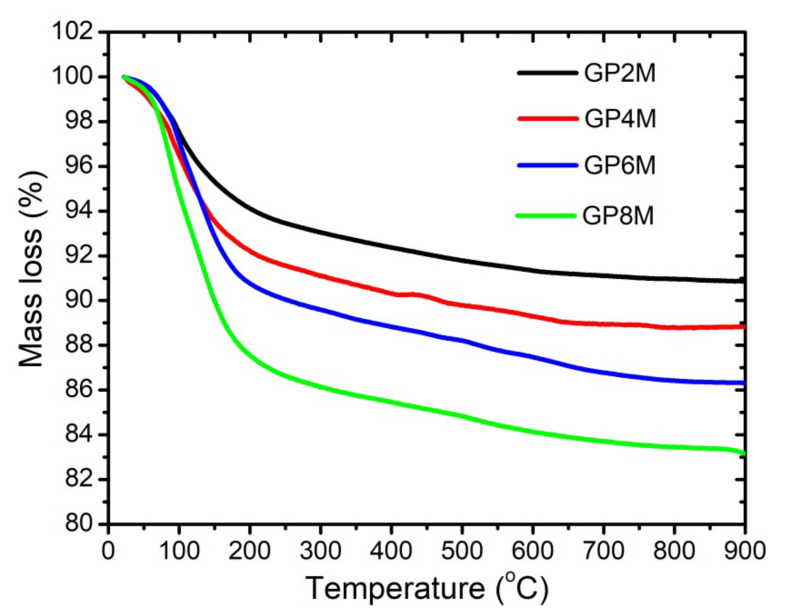
TGA of the reference geopolymer samples GP2M–GP8M.

Figure 2 shows the thermogravimetric curves of GP2M–GP8M samples. Water evaporation and dihydroxylation are probably the effects liable for the loss mass during heat treatment of geopolymer. Physical and chemical water from hardened geopolymers evaporates at 20∼100 °C (region I) and 100∼300 °C (region II), respectively. At temperatures above 300 °C, hydroxyl groups evaporated (region III) [5,21]. The total mass loss for all investigated geopolymer samples varies from about 8 to 16% at 900 °C.

The mass loss is ~4, 6, 8, and 12% in the second region for GP2M, GP4M, GP6M, and GP8M, respectively. Due to heat treatment in the temperature region from 300 °C up to 850 °C (region III) of geopolymer samples, the process of condensation of silanol (Si-OH) and aluminol groups (Al-OH) of the geopolymer gel occurs and Si-O-Si or Si-O-Al bonds are created. The weight loss in this region is between 1.0% and 3.0%. The mass loss at over 750 °C happens due to the decomposition of carbonate species.

**Figure 3:**
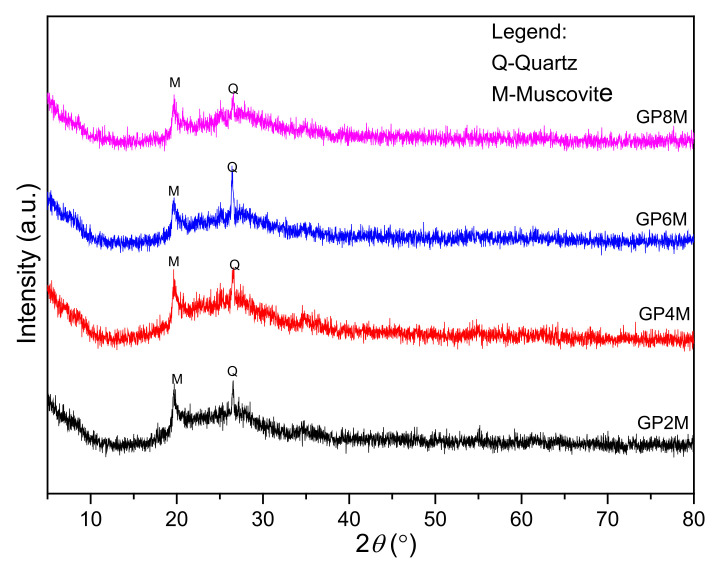
XRD results of samples: GP2M, GP4M, GP6M, and GP8M.

At a higher temperature of 800–850 °C, reaction of sintering starts, leading to the creation of ceramic material [21,22,23,24]. Low mass loss over 800–850 °C (region IV) indicates the stopping of additional thermal decomposition of geopolymer samples [19,20]. Over 70% of the total weight loss is observed below 300 °C (Figure 2), which suggests that most of the water in the geopolymer materials is present as water that evaporates openly. The residual water is present as adsorbed water in many different pores of surface of geopolymer gel. Some researchers have detected that the nepheline phase is formed when the geopolymers are heated to 1000 °C [15,19]. They concluded that the weight loss at high temperatures is connected to the creation of nepheline phases. In other words, nepheline contained in geopolymer mortar is a consequence of the weight loss after geopolymer exposure heating up to 750 °C [25]. In our case, the nepheline phase (Figure 3) was identified on the thermally treated geopolymer samples. Additionally, sanidine is formed at high temperature. This means that their formation affects changes in mass, as well as energy changes in the system. On the DTA thermogram, the changes in the structure of geopolymer samples resulting from the elimination of hydroxyl groups are poorly visible. The reason is very slow processes that lead to their elimination in the wide temperature interval examined [23,24].

### 2.2. Structural and Chemical Characterization of Referent and Thermally Treated Geopolymer Samples

#### 2.2.1. XRD Analysis

Using XRD analysis, the measurement of structural and phase analysis of geopolymer samples was performed. X-ray diffraction patterns of GP_Refs_-GP2M (a), GP4M (b), GP6M (c), and GP8M (d) are shown in Figure 3. X-ray diffraction analysis of all geopolymer samples shows the formation of an amorphous phase of the matrix indicated by the high baseline in the range between 18° and 34° 2*θ*, which indicates an amorphous structure with impurities of the crystalline phase SiO_2_ (α-quartz), which also occurs in metakaolin [26,27]. Additionally, the presence of muscovite was observed in all GP_Ref_ samples. In general, the activation of metakaolin by different concentrations of alkaline activators does not change the mineral composition of geopolymer samples.

**Figure 4:**
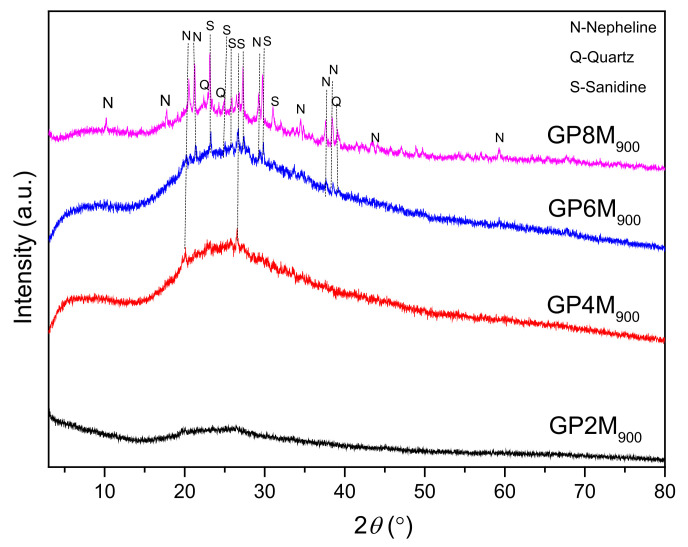
XRD results of thermally treated geopolymer samples: GP2M_900_, GP4M_900_, GP6M_900_, and GP8M_900_.

After thermal treatments, X-ray diffractograms were recorded (Figure 4); after treatments at 900 °C, the diffractogram of the GP2M_900_ sample dominantly shows the presence of amorphous phases, while the diffractograms of other samples besides the amorphous phase consist of crystal phase of quartz (GP4M_900_), sandine and nepheline (GP6M_900_ and GP8M_900_). Qualitatively, it is observed that the amount of the amorphous phase increases with the concentration of NaOH. Nepheline and sanidine are minerals that belong to the group of feldspar and in a group of potassium sodium aluminum silicate compounds [28]. Due to thermal treatment, muscovite decomposes at temperatures up to 800 °C, to high-temperature phases. Sodium and potassium are always present in naturally occurring kaolinite and the addition of NaOH with a high concentration to metakaolin and heating at a temperature of 900 °C leads to the crystallization of potassium sodium aluminum silicate compounds, i.e., compounds of the type corresponding to nepheline and sanidine by the literature [28]. So artificially prepared materials have the composition (Na, K) AlSiO_4_, which represents the true composition of nepheline. Low-intensity quartz peaks belong to α-quartz, which crystallizes at temperatures between 573 and 870 °C [28]. The formation of nepheline and sanidine based on the results is in a direct branch with the used molarity of NaOH solution. The addition of Na, Al, and Si from high-molality activator reagents leads to the recrystallization of new compounds at high temperatures.

#### 2.2.2. FTIR Analysis

**Figure 5:**
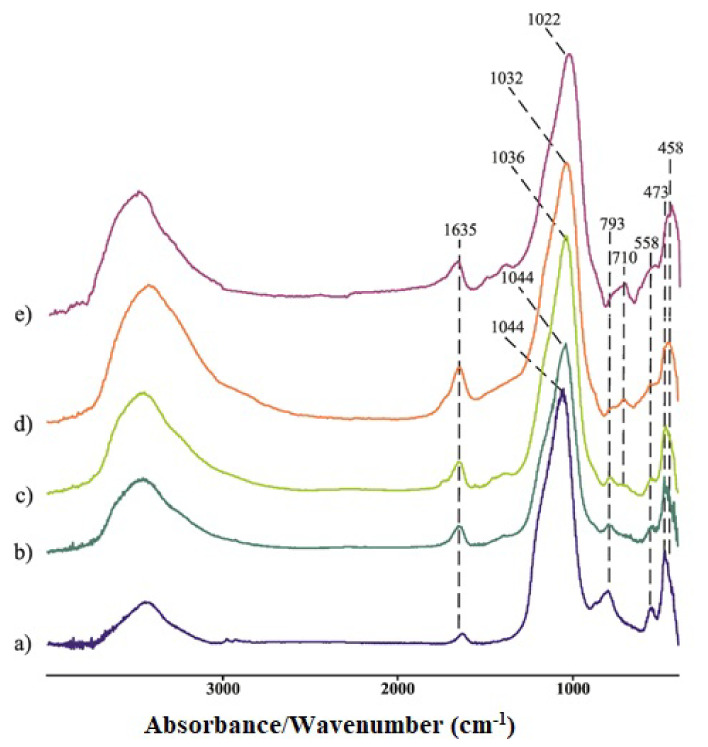
FTIR spectra of metakaolin (**a**) and the geopolymer samples GP2M-(**b**), GP4M-(**c**), GP6M-(**d**), and GP8M-(**e**).

Figure 5 shows the FTIR spectra reference geopolymer samples. The spectra of the samples were collected in the 4000–400 cm^−1^ region.

The FT-IR spectra of the metakaolin (MK) and reference geopolymer samples show broad bands at ~3500 cm^−1^ due to O–H and H–O–H stretching vibrations. H–O–H bending vibrations are observed in spectrum at 1635 cm^−1^ [29,30]. Sharp and strong bands at 1044, 1036, 1032, 1022 cm^−1^ for GP2M, GP4M GP6M and GP8M, respectively, appear due to Si–O stretching modes [30]. Additionally, an area of approximately 1050 cm^−1^ can be attributed to the existence of asymmetric Si–O–Si and Al–O–Si stretching vibrations, which are the building blocks of the geopolymer gel. The difference in position of bands is not great, but it still exists. It has been observed that increasing NaOH concentration, the values of wavenumber of this vibration shifts towards lower values and may be attributed to the partial replacement of silicon–oxygen tetrahedron by AlO_4_ tetrahedron [30]. The band at wavenumber 793 cm^−1^ can be related to symmetric stretching vibrations of Si–O–Si. Bands that appeared at wavenumbers region 550–710 cm^−1^ were also a consequence of the symmetric stretching vibrations of Si–O–Si and Al–O–Si. Bending vibrations of Si–O–Si and O–Si–O are associated with wavenumbers between 450 and 480 cm^−1^ [8,31].

**Figure 6:**
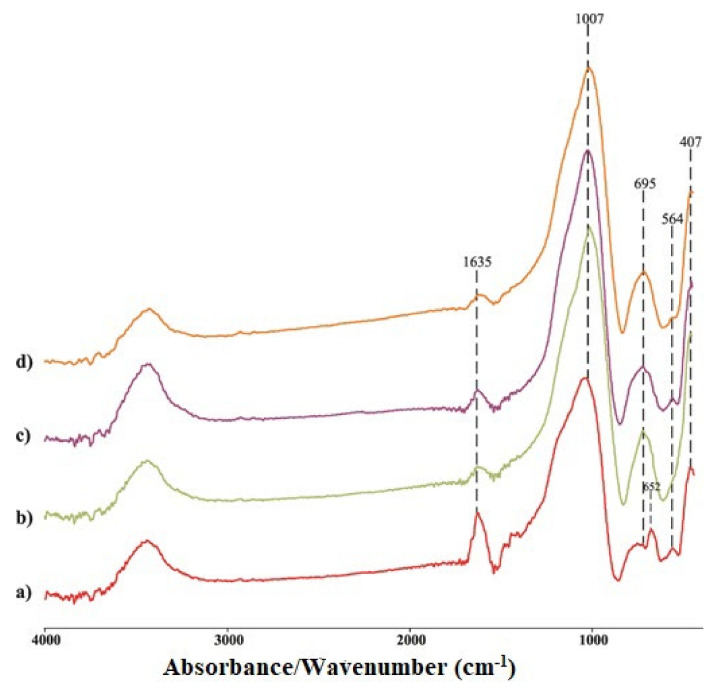
FTIR spectra of thermally treated geopolymer samples; GP2M_900_-(**a**); GP4M_900_-(**b**); GP6M_900_-(**c**); GP8M_900_-(**d**).

Figure 6 shows FTIR analysis of thermally treated GP_Ref_ samples from 30 to 900 °C. As with the GP_Ref_ samples, vibration bands are observed at about 3440 cm^−1^ and 1635 cm^−1^.

The FTIR band at 1007 cm^−1^ corresponds to Si-O or Si-O-M (M-is Si or M or OH) elongation of a tetrahedron in which Si is surrounded by three oxygen bridges and one non-bridging oxygen (NBO) [32,33,34]. The position of this bond is identical for all investigated thermally treated geopolymer samples. The change in band position at this wavenumber of GPnM_900_ samples compared to GP_Ref_ may be related to an enhancement in the proportion of Si with NBO atoms in thermally treated samples. This shift can be attributed to an increase in the proportion of Si with NBO atoms [35]. In our case, there is an obvious shift in the elongation range Si-O-M relative to untreated samples. Additionally, in all thermally treated samples, new peaks appear at 695, 564, and 407 cm^−1^, which indicates that structural changes occur during thermal treatment. Therefore, GPnM_900_ samples appear to have a moderately regulated structure, while annealing increases the bond strength, thus improving mechanical properties and creating new ceramic-like materials [14].

According to the literature [36], two bands at 793 cm^−1^ and 713 cm^−1^ (GP2M–GP8M) can be related to the asymmetric stretching of Si-O-Al bonds. These bands were not detected in the heat-treated samples. Vibration maxima at 558 cm^−1^ for the GP_Ref_ samples were displaced to a larger angle for the heat-treated samples. Then, low-intensity vibration maxima at about 473 cm^−1^ do not occur with heat-treated samples. Additionally, the vibration maximum at 458 cm^−1^ in GP_Refs_ was shifted to 407 cm^−1^ in heat-treated samples. The formation of nepheline observed through XRD was confirmed by the appearance of peaks around 695 cm^−1^ and a shoulder between 1000 and 1100 cm^−1^ [37] for GP4M_900_, GP6M_900_ and GP8M_900_ geopolymer samples in the FTIR spectra.

#### 2.2.3. Morphological Analysis

**Figure 7:**
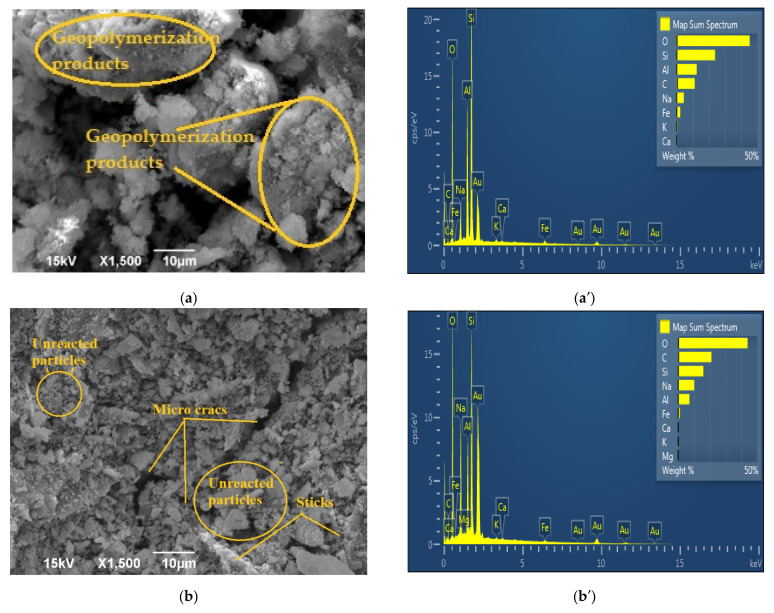

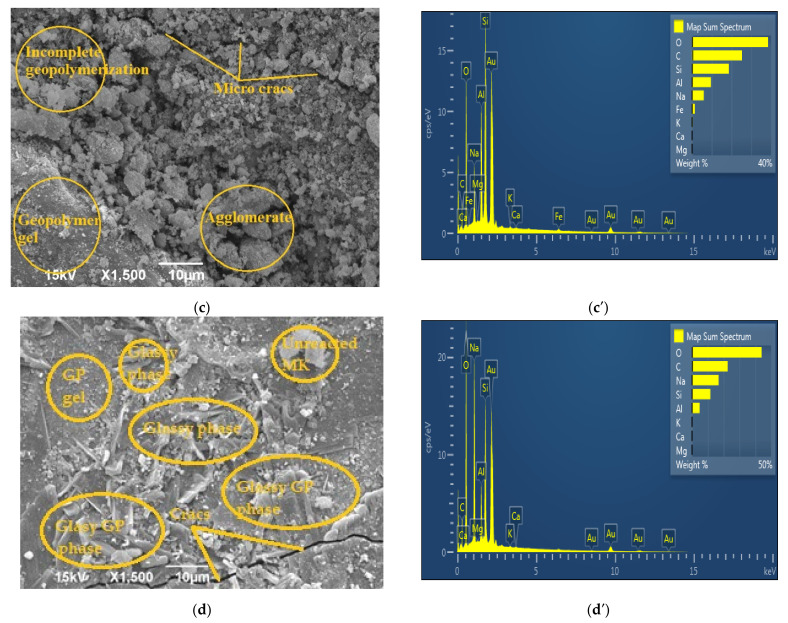
SEM micrographs of reference geopolymer samples: (**a**) GP2M; (**b**) GP4M; (**c**) GP6M; (**d**) GP8M; EDS spectrum of (**a′**) GP2M; (**b′**) GP4M; (**c′**) GP6M; (**d′**) GP8M.

The surfaces of the GP_Ref_ samples are shown in Figure 7a–d as well as the EDS graphs corresponding to these surfaces. The surface of all investigated GP_Ref_ samples is porous and consisted of some unreacted particles and a geopolymer matrix formed during polymerization–geopolymerization. The alkaline activator solution decomposes metakaolin, releasing Si^4+^ and Al^3+^ ions, which then participate in the geopolymerization reaction. A geopolymer gel is formed, which indicates that there is a change in the structure of raw materials. Figure 7a shows a micrograph of the GP2M sample. The microstructure of geopolymer obtained by alkali activation metakaolin with alkali activator that contains 2 mol dm^−3^ NaOH is more fragile compared to other samples. A structure is observed, which is probably a consequence of the incomplete process of dissolving metakaolin, i.e., incomplete geopolymerization. Figure 7b shows individual particles, aggregates, and gel phases, as well as formed rods of the GP4M sample. The structure of the GP6M is somewhat more compact with a small number of single particles and a larger proportion of grouped particles (Figure 7c). The GP8M porous gel structure is shown in Figure 7d. There are also several individual particles. Based on the presented microstructures GP2M, GP4M, and GP6M (Figure 7a–c), inter granular cracks are observed. SEM analysis of GP8M (Figure 7d) shows that the microstructure is a densely packed plate structure. Additionally, on the surface of the samples (Figure 7b–d), micro cracks are observed. They are especially manifested in the GP4M sample (Figure 7b).

**Figure 8:**
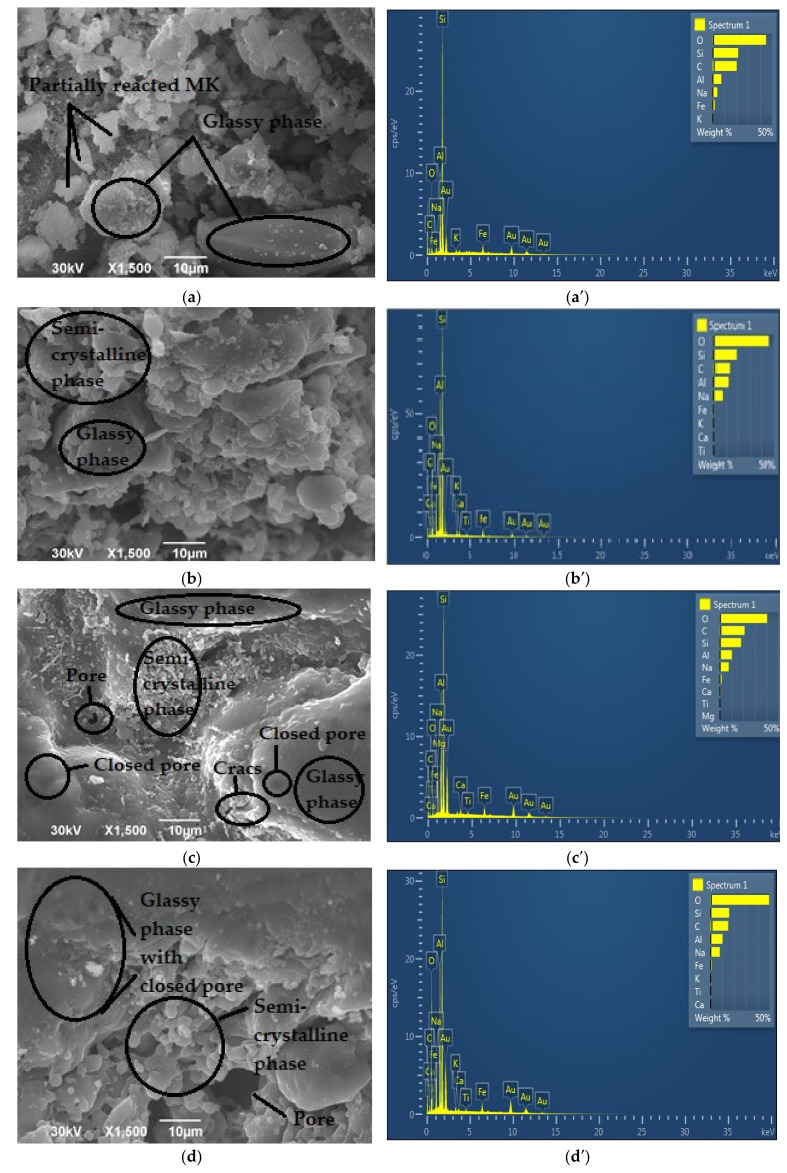
SEM micrographs of thermally treated geopolymer samples: (**a**) GP2M_900_; (**b**) GP4M_900_; (**c**) GP6M_900_; (**d**) GP8M_900_; EDS of thermally treated geopolymer samples: (**a′**) GP2M_900_; (**b′**) GP4M_900_; (**c′**) GP6M_900_; (**d′**) GP8M_900_.

The microstructure of geopolymers annealed at 900 °C is shown in Figure 8a–d. After exposure of the materials at 900 °C, a more homogeneous matrix was obtained, due to the partial melting of the geopolymer matrix. GP_Ref_ samples are partially melted and then hardened, creating quite small pores in the sample, while the material is transformed from an amorphous geopolymer gel into a new amorphous phase and nepheline and sanidine semi-crystalline ceramics (according to XRD analysis). Phase analysis of GP2M_900_ sample determined that its structure is almost completely amorphous. Additionally, small quartz particles are visible on the surface of the sample, which was confirmed by XRD analysis. Comparison of SEM micrographs of thermally treated geopolymer samples (Figure 8a–d) showed different morphologies. In the GP2M_900_ sample, an inhomogeneous pore structure with individual crystalline structures of a different shape is observed. The pores are randomly distributed. The difference in the microstructure of the reference sample and the thermally treated samples is a consequence of the defined experimental conditions, which include the controlled heating rate and the processes that take place during the heating of the inorganic polymer in the airflow atmosphere. In GP4M_900_, a glassy structure of geopolymer gel with smaller pores, cracks, and also unreacted small particles is observed. GP6M_900_ and GP8M_900_ have a higher proportion of crystalline phases (quartz, nepheline and sanidine, Figure 4), but the structure is still quite porous and the observed crystals are irregular and varied in shape (Figure 8c,d).

Table 1 shows the ratios Si/Al and Si/Na, based on EDS analysis of the displayed areas of all tested samples.

From Table 1, we can see that the Si/Na ratio decreases in reference geopolymer samples due to the increase in NaOH concentrations, which was expected. Additionally, we can see that the Si/Al ratio increases with increasing concentration, but that it is less than 3.0 for all samples, which is very conducive to good mechanical properties. As the pH increases, more silicon is dissolved from MK, but at the same time, the reaction of calcium, which is present as an impurity in the raw material, is prevented. Increasing the pH of the alkaline activator solutions inhibits the release of calcium into the system, building the calcium silicate hydrate phase. Calcium oxide exists as an impurity in metakaolin as a precursor. Geopolymer gel is created as the main phase. As for the thermally treated samples, the Si/Al ratio is slightly lower than the reference ones, which is understandable because during the thermal treatment up to 900 °C, the existing structure decomposes, and the compounds Si-OH and Al-OH evaporate. A similar dependence is observed for the Si/Na ratio.

### 2.3. Compressive Strength of Geopolymer Samples

Compressive strength is a very important mechanical property that affects the quality and applications of materials. The higher concentration of alkali in the alkaline activator during the geopolymerization process leads to the creation of products with high structural integrity. Data from the literature show that the formation of carbonates negatively affects the strength of geopolymers [38]. The lack of Si and Al species after dissolving metakaolin in alkali activation solutions influences a geopolymer matrix that has poor mechanical characteristics. Alkaline ion deficiency could affect the participation of Al ions, because the alkaline ion is needed to maintain the charge balance [39,40]. The Si/Al ratio is the parameter that most affects the compressive strength of geopolymer samples. The addition of silicon through solution activation favors the synthesis of geopolymers with improved mechanical performance. Additional Si ions affect the formation of the geopolymer matrix by acting as nucleation sites for the polycondensation reaction. If the Si/Al ratio increases above 3.0, it negatively affects the compressive strength. The reason is that the excess of Si ions in the activation solution prevents the dissolution of the aluminosilicate precursor. The particle size of MK as a precursor is an important factor for geopolymer creation. A higher percentage of particles of different sizes, and some percent of unreacted particles of MK mean that there is most likely an uneven distribution and accumulation in some places, which becomes critical because the amount of amorphous phase is smaller, and thus so is the amount of amorphous gel. These are potentially critical points where the matrix–amorphous gel cracks due to overload with accumulated unreacted particles. All these phenomena must be taken into account when interpreting the compressive strength of geopolymers.

**Figure 9:**
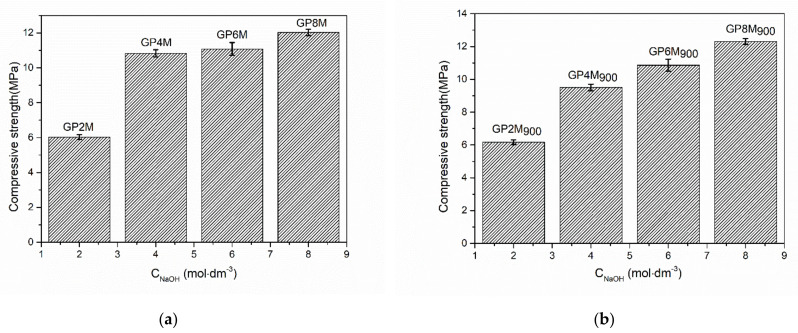
(**a**) Compressive strength of the geopolymer samples GP2M−GP8M; (**b**) Compressive strength of the heat-treated geopolymer samples GP2M_900_−GP8M_900_.

Figure 9 shows the compressive strength of the reference GP2M–GP8M (a) and heat-treated samples GP2M_900_−GP8M_900_ (b). It can be observed that the values of compressive strength of GP_Ref_ samples increase, but for the geopolymer samples GP4M-GP8M, the values are approximately the same, especially for GP4M and GP6M. MK-based geopolymer showed that the best conditions of geopolymerization to develop a higher compressive strength of 20 MPa using 12 M KOH were 60 °C and 28 curing days [41]. The value of the compressive strength of MK geopolymers obtained using 8M NaOH as part of the alkaline activator is slightly lower than the value obtained in the above-mentioned research [41]. Some reseachers found that heat curing was also necessary to make a rapid geopolymerization reaction to realize a sustainable strength within short periods, but in the others mentioned, a higher curing temperature does not automatically mean that the compressive strength of the investigated samples will be higher.

The strength of MK geopolymers decreases significantly after exposure of geopolymers to high temperatures. As the temperature rises, water, in all its forms, as well as other volatile compounds in geopolymers begin to evaporate. MK geopolymer matrix is dense even at elevated temperatures. However, the vapor pressure at the pore boundaries is constantly rising during heating because there is no channel for the vapor pressure dissipation. For this reason, when the vapor pressure reaches a maximum, the geopolymer matrix cannot stand the thermal stress. This leads to the creation of cracks in the matrix. Tests show that MK-based geopolymers undergo significant thermal shrinkage and high strength reduction after thermal treatment up to 800 °C [42]. That is why programmed and slow heating during thermal treatment is very important. The presence of quartz as an impurity in the matrix can maintain the stability of the geopolymer at high temperatures [43]. Geopolymers based on metakaolin with fine particles have higher compressive strength after heat treatment at 1000 °C since fine particles serve as fillers in the gap between larger aggregates [44]. Therefore, fine particles that are incorporated into the structure in an above-mentioned manner create an effective barrier to stress or deformation, which prevents the fracture of the geopolymer matrix during thermal treatment at high temperatures. The values of the compressive strength of the geopolymer thermally treated up to 900 °C are close but slightly lower than the untreated geopolymer [45]. Thermal treatment of geopolymer samples in this temperature range caused the concentration of the geopolymer matrix. This process is similar to the process of viscous sintering because the geopolymer amorphous network softens while the space between the particles in the geopolymer gel is destroyed [46]. Some of the previous studies related to the examination of the properties of MK geopolymers at high temperatures [42,47] show that the strength of MK geopolymers decreases significantly after exposure to high temperatures. As the temperature increases, the water and the other volatile compounds in the geopolymer samples begins to evaporate. The vapor pressure on the porous walls is constantly increasing due to the condensing of the matrix, so there is no possibility to release the vapor pressure in the matrix. Therefore, when the vapor pressure reaches a maximum, the dense matrix cannot withstand the high thermal stress, which leads to the growth of cracks and, in some cases, to the complete destruction of the structure [5]. The conditions of thermal treatment are very important because it is enough that a change in only one parameter, such as the heating rate, causes large changes in the structure of geopolymer samples.

Up to 100 °C, structural elasticity is preserved because only free water is lost in this interval. The geopolymer treated to these temperatures is dimensionally stable and dense as before the treatment. Further dehydration (100–300 °C) caused by the expulsion of water from micro- and nano-pores effects the dimensional reduction and deformation of geopolymer. In the temperature range of 300–800 °C, a higher shrinkage was observed compared to the previous phase. In this temperature range, condensation occurs between Si-OH and Al-OH groups. When the geopolymer is thermally treated above 800 °C, a viscous sintering process occurs. Dimensional reduction in this phase is caused by the creation of a molten amorphous glass phase [44,48]. Practically, at temperatures above 800 degrees, the process of sintering starts, and thus, the density as well as the strength of the material increases. Figure 9b shows the dependence of thermally treated geopolymer samples up to 900 °C as a function of the NaOH molarity as part of the alkaline activator. Based on the obtained results, an increase in strength with an increase in concentration of alkaline activator is observed. The values of the obtained compressive strengths are slightly higher than the strength of GP_Ref_ samples. Given that up to 800 °C there is a deterioration of the structure, melting of the geopolymer matrix and large structural changes in the geopolymer, it is understandable that a small increase in the compressive strength of the geopolymer would occur after finishing thermal treatment up to 900 °C. The temperature range of 800–900 °C is practically a transitional temperature area. In general, for thermally treated GP_Ref_ and GP, the obtained values of the compression strength for the applied system, metakaolin precursor and alkaline activator formulation as well as temperature and storage polymerization conditions are of great benefit for future experiments.

## 3. Conclusions

The present study ensured access to the properties and thermal stability of metakaolin-based geopolymer samples in terms of mass loss, thermal deformation, structural changes and change in compressive strength of geopolymer samples exposed to high/transition temperature.

- The XRD results of all investigated GP_Ref_ samples show almost the same mineralogical contents. GP_Ref_ samples consist of an amorphous structure with quartz and muscovite as an impurity. After heating GP_Ref_ until 900 °C, new semi-crystalline phases appeared in GP4M_900_–GP8M_900_.
- FTIR analysis of both groups of samples confirmed the formation of a new amorphous gel with a certain proportion of crystalline phases after heating. The shift of wavenumbers towards higher or lower values is an indicator of these changes. Additionally, some bands do not appear in GPnM_900_, as a consequence of the process of dehydroxylation and further condensation.
- The microstructure of geopolymer samples treated thermally up to 900 °C was significantly changed in relation to the microstructure of GP_Ref_ samples. Samples GP6M_900_ and GP8M_900_ are more compact than others and crystallites are observed, as is the predominant dense glass matrix. The effect of alkaline activator concentration is visible in both GP_Ref_ samples and GPnM_900_ samples.
- Based on the DTA results, it can be concluded that among the examined geopolymers, there are small differences which occur during thermal treatment, which are most likely a function of the structural composition as well as thermal stability of geopolymers.
- The compressive strength of the thermally treated geopolymer samples increases as a function of the concentration of NaOH as a component of an alkali activator solution. There are no significant changes in the corresponding values for the compression strength of GP_Ref_ samples and GPnM_900_.
- Given the obtained results, we continue research in the direction of further increasing the temperature of thermal treatment, as well as other conditions that may affect the change in compressive strength.

## 4. Materials and Methods

Metakaolin (MK) was used as a precursor of geopolymer samples. Metakaolin (MK) was prepared by calcining kaolinite clay [49] (originated from Serbia) at 750 °C for one hour. Alkaline activator solutions were made by mixing Na_2_SiO_3_ and NaOH in an appropriate volume ratio. The concentration of NaOH (Sigma Aldrich, analytical grade) solution was 2, 4, 6, and 8 mol dm^−3^. Metakaolin and alkali activator solution were mixed for 20 min at room temperature. The ratio of solid and liquid phase was approximately 1:1. The mixtures were cast into circle plastic molds (20 × 40 mm). The samples were stored under laboratory conditions for one day in covered molds to prevent water evaporation. After that, the samples were put in a sample drying oven for two days at 60 ± 1 °C. Finally, the samples were put in a climate chamber in a controlled condition and aged for twenty-eight days. These samples of geopolymers are marked as GPnM (n is the molarity of a solution of NaOH). These samples, GP2M–GP8M, represent reference samples (GP_Ref_). These geopolymers were heated in a furnace in an air atmosphere from 30 to 900 °C at a heating rate of 1 °C min^−1^ to investigate changes in their structural properties. The samples were stored at the given temperature for one hour and then spontaneously cooled to room temperature. The thermally treated GP_Ref_ samples are denoted as GPnM_900_ (n-molarity of solution of NaOH).

The thermal stability of the samples was determined by simultaneous TG-DTA (Setsys 2400 CS Evolution, SETARAM Instrumentation, Caluire, France) in the temperature range between 30 and 900 °C under the airflow of 20 mL·min^−1^. All samples were characterized at room temperature by X-ray powder diffraction (XRPD) using an *Ultima IV* Rigaku diffractometer, equipped with Cu Kα_1,2_ radiation, using a generator voltage (40.0 kV) and a generator current (40.0 mA). The range of 5–80° 2*θ* was used for all powders in a continuous scan mode with a scanning step size of 0.02° and at a scan rate of 5°/min. For phase analysis, PDXL2 software was used equipped with the ICDD database [50,51]. PDF card numbers for *α*-quartz (01-089-8937), muscovite (01-080-0743), nepheline (01-073-6265), sanidine (01-0860101).

The functional groups of all samples were studied using FTIR spectroscopy at room temperature using a Bomem (Hartmann & Braun, Frankfurt am Main, Germany) MB-100 spectrometer. The microstructure analysis of the obtained GP materials was performed using a JEOL JSM 6390 LV electron microscope at 25 kV. The composition of MK was determined by means of X-ray fluorescence analysis (XRF). Table 2 shows the chemical composition of metakaolin made by the XRF method (type UPA KSRF 200).

Compressive strength was performed on a HPN400 type press (ZRMK-Ljubljana, Ljubljana, Slovenia). For each reference and heat-treated samples, a set of three specimens was used.

## Figures and Tables

**Table 1:** The ratios Si/Al, Si/Na, based on EDS analysis of the displayed areas of all investigated samples.

Sample	GP2M	GP4M	GP6M	GP8M	GP2M_900_	GP4M_900_	GP6M_900_	GP8M_900_
Si/Al	1.910	1.930	1.960	2.090	1.710	1.770	1.630	1.580
Si/Na	5.283	3.146	2.670	2.521	4.651	2.398	2.435	2.005

**Table 2:** Chemical composition of metakaolin by XRF method.

Oxide	SiO_2_	Al_2_O_3_	Fe_2_O_3_	MgO	CaO	Na_2_O	K_2_O	*LoI
% wt	53.03	35.44	4.39	1.25	1.38	0.01	2.06	0.44

* loss of ignition.

## Data Availability

We exclude this statement because we don’t have any data.
